# The European Perspective and History on Biosimilars for the Treatment of Inflammatory Bowel Diseases

**DOI:** 10.1093/crocol/otab012

**Published:** 2021-02-23

**Authors:** Virginia Solitano, Ferdinando D’Amico, Leonardo Da Rio, Laurent Peyrin-Biroulet, Silvio Danese

**Affiliations:** 1 Department of Biomedical Sciences, Humanitas University, Pieve Emanuele, Milan, Italy; 2 Department of Gastroenterology and Inserm NGERE U1256, University Hospital of Nancy, University of Lorraine, Vandoeuvre-lès-Nancy, France; 3 Department of Gastroenterology, IBD Center, Humanitas Clinical and Research Center, IRCCS, Rozzano, Milan, Italy

**Keywords:** biosimilars, inflammatory bowel diseases, switching, Europe

## Abstract

With the expiration of biologics’ patents, biosimilars entered the market as a promising opportunity to reduce health-care costs in the field of inflammatory bowel diseases. Although biosimilars were initially poorly accepted, the growing evidence about their efficacy and safety has changed this situation, resulting in their widespread use. However, there is still an unmet need of improving patients’ education about biosimilars to minimize nocebo responses and to accept nonmedical switching. Looking to the future, the use of recently authorized adalimumab biosimilars and the first attempts of adopting different strategies of switching (eg, cross-, multiple-) will fill some residual knowledge gaps.

## INTRODUCTION WITH DEFINITIONS

Biologics have revolutionized the management of inflammatory bowel diseases (IBDs). However, the development process of these drugs is extremely expensive and time-consuming. The Crohn’s & Colitis Foundation identified biologics as the key driver of health-care costs for patients with IBD, highlighting the crucial importance for enacting cost-effective strategies.^1^ In this context, biosimilars are defined by the European Medicines Agency (EMA) as medications that are “highly similar” to their originators; they entered the market with the intent of lessening health-care expenses, thus increasing the accessibility of biopharmaceutical treatments to a wider number of patients.^2^ Hence, the term “nonmedical switching” was coined to indicate a change in a patient’s drug (eg, switching from an originator biologic to its biosimilar), which is not due to medical reasons, but generally to cost savings. The EMA approval process relies on stringent, though accelerated, regulatory standards that involve a so-called comparability exercise of physiochemical characteristics, efficacy, safety, and immunogenicity.^2^ The authorization of a biosimilar for clinical indications of the originator without the need to conduct clinical trials in these other indications is defined as extrapolation. It allows the comparison of clinical data across different indications.^2^ The term “interchangeability” refers to the possibility of replacing one drug with another that is considered to have the same clinical properties, by either switching or automatically substituting compounds.^2^ Whether the originator can be switched or substituted with the biosimilar is not regulated by EMA, but the decisions are made at the national level. Subsequently, not in all European countries, the prescription of a biosimilar instead of the originator by either the prescriber (switching) or the pharmacists (automatic substitution) is permitted. For instance, in Italy and Germany only the prescribing physician can assume the responsibility for administering either an originator or its biosimilar after an affordability assessment, whereas France was the first European Union (EU) member to approve biosimilar substitution for those who were naive to a biological drug.

## TIMELINE OF EUROPEAN HISTORY AND PERSPECTIVES ON INFLIXIMAB AND ADALIMUMAB BIOSIMILARS

Looking to the past, before the introduction of biosimilars in clinical practice, physicians exhibited a cautious and conservative approach. An ECCO survey conducted in 2013 revealed that more than 60% of clinicians were concerned about prescribing biosimilars.^3^ In the same year, the first biosimilar agent of infliximab, CT-P13, was approved by the EMA for IBD treatment, based on the extrapolated data of two pivotal trials conducted in rheumatologic diseases (PLANETAS and PLANETRA).^4,5^ Afterward, a multitude of studies enrolling patients with IBD reported the noninferiority of CT-P13 compared with the infliximab originator^6^ and an updated ECCO survey indicated that only 20% of physicians still felt some uncertainty toward biosimilars after 2 years since their market entrance.^7^ Interestingly, in 2015, the results of a questionnaire from the European Federation of Crohn’s and Ulcerative Colitis Association showed that patients’ perspectives reflected the initial clinicians’ skeptical viewpoint, as almost half of the respondents were worried about the intrinsic biosimilars’ properties.^8^ In 2016, SB2, a second infliximab biosimilar, received marketing authorization in Europe. Even then, the EMA approval was based on clinical equivalence studies in patients with rheumatological disorders.^9,10^ In 2017, the PROSIT-BIO and the PROSIT follow-up studies demonstrated that the safety and efficacy of CT-P13 were comparable with the reference product in a large cohort of patients with IBD.^11,12^ The NOR-SWITCH trial randomized 482 patients to be treated with the infliximab originator or to be switched to CT-P13.^13^ The adjusted treatment difference between the two arms was within the established cutoff of 15%, confirming the noninferiority of CT-P13. In the meantime, data regarding price reduction in the European Economic Area were soon made public; according to a report by QuintilesIMS published in 2017, biosimilar competition had already reduced average list prices (prices per treatment day in 2016 vs the year before biosimilar introduction from −2% to −39%) and significantly increased access to biological medicines for patients.^14^ In 2018, another infliximab biosimilar, PF-06438179/GP111, was authorized in Europe and the patent of adalimumab reference product expired, leading to the approval of several biosimilars soon afterward. Regulatory approval of adalimumab biosimilars for IBD treatment was based on data from several trials on patients with rheumatoid arthritis and plaque psoriasis.^15^ Data on the efficacy and safety of adalimumab biosimilars and new strategies of switching (eg, reverse, multiple, and cross-switch) have recently become available. A 2019 retrospective study enrolling patients with IBD who had switched from the originator adalimumab to the biosimilar SB5 did not show any meaningful difference in terms of clinical, laboratory, and pharmacokinetic outcomes between SB5 and the originator drug.^16^ Moreover, a multicenter prospective study provided first evidence regarding the safety and efficacy of the infliximab biosimilar SB2, also including a minority of patients who had cross-switched from CT-P13 to SB2 and multiple-switched from the infliximab originator to CT-P13 and then to SB2.^17^ Despite reassuring findings, there is still evidence of a lack of familiarity and persisting concerns about the use of biosimilars among patients.^18^ Reluctance and negative expectations toward switching to biosimilars are believed to contribute to the worsening or onset of symptoms, generally referred to as nocebo effect. For this reason, health-care workers should provide reassuring information about biosimilars and their properties, through an empathic and powerful communication, to minimize nocebo responses.^19^ The experience acquired so far has paved the way toward an increased knowledge and confidence in the proven efficacy and safety of biosimilars. Over the past 7 years, no safety issues have emerged related to the use of biosimilars.

## FUTURE DIRECTIONS

Recently, a new subcutaneous formulation of CT-P13 showed promising results in phase I and II trials in both Crohn’s disease and ulcerative colitis.^20,21^ Clinical efficacy and safety were comparable and serum trough levels appeared to be more stable than conventional intravenous CT-P13. If these preliminary results will be confirmed in phase III trials (NCT03945019 and NCT04205643), the first subcutaneous version of infliximab could soon be available, enriching the therapeutic armamentarium for patients with IBD. In addition, ongoing studies (NCT04422171 and NCT03662919) will provide further information about adalimumab biosimilars and alternative strategies of switching. More patents are about to expire (eg, certolizumab in 2021, golimumab and vedolizumab in 2024), and the development pipeline of many biosimilars raises new doubts regarding the choice of the biosimilar. Head-to-head comparative trials between the different biosimilars of the same originating molecule are eagerly awaited to evaluate whether one biosimilar is superior to the other.

## Data Availability

Data sharing is not applicable to this article, as no new datasets were generated or analyzed during the current report.

## Notes

Funding: No funding.

Conflict of Interest: V. Solitano, F. D’Amico, and L. Da Rio declare no conflict of interest. L. Peyrin-Biroulet has served as a speaker, consultant, and advisory board member for Merck, AbbVie, Janssen, Genentech, Mitsubishi, Ferring, Norgine, Tillots, Vifor, Hospira/Pfizer, Celltrion, Takeda, Biogaran, Boerhinger-Ingelheim, Lilly, HAC Pharma, Index Pharmaceuticals, Amgen, Sandoz, Forward Pharma GmbH, Celgene, Biogen, Lycera, Samsung Bioepis, and Theravance. S. Danese has served as a speaker, consultant, and advisory board member for Schering-Plough, AbbVie, MSD, UCB Pharma, Ferring, Cellerix, Millenium Takeda, Nycomed, Pharmacosmos, Actelion, Alphawasserman, Genentech, Grunenthal, Pfizer, Astra Zeneca, Novo Nordisk, Cosmo Pharmaceuticals, Vifor and Johnson & Johnson, Nikkiso Europe GmbH, and Theravance.
